# The Neutrophil: Constant Defender and First Responder

**DOI:** 10.3389/fimmu.2020.571085

**Published:** 2020-09-24

**Authors:** Noah Fine, Nikola Tasevski, Christopher A. McCulloch, Howard C. Tenenbaum, Michael Glogauer

**Affiliations:** ^1^Faculty of Dentistry, University of Toronto, Toronto, ON, Canada; ^2^Centre for Advanced Dental Research and Care, Mount Sinai Hospital, Toronto, ON, Canada; ^3^Department of Dental Oncology, Maxillofacial and Ocular Prosthetics, Princess Margaret Cancer Centre, Toronto, ON, Canada

**Keywords:** neutrophil, mucosal immunity, dybiosis, PMNs, inflammatory disease

## Abstract

The role of polymorphonuclear neutrophils (PMNs) in biology is often recognized during pathogenesis associated with PMN hyper- or hypo-functionality in various disease states. However, in the vast majority of cases, PMNs contribute to resilience and tissue homeostasis, with continuous PMN-mediated actions required for the maintenance of health, particularly in mucosal tissues. PMNs are extraordinarily well-adapted to respond to and diminish the damaging effects of a vast repertoire of infectious agents and injurious processes that are encountered throughout life. The commensal biofilm, a symbiotic polymicrobial ecosystem that lines the mucosal surfaces, is the first line of defense against pathogenic strains that might otherwise dominate, and is therefore of critical importance for health. PMNs regularly interact with the commensal flora at the mucosal tissues in health and limit their growth without developing an overt inflammatory reaction to them. These PMNs exhibit what is called a para-inflammatory phenotype, and have reduced inflammatory output. When biofilm growth and makeup are disrupted (i.e., dysbiosis), clinical symptoms associated with acute and chronic inflammatory responses to these changes may include pain, erythema and swelling. However, in most cases, these responses indicate that the immune system is functioning properly to re-establish homeostasis and protect the status quo. Defects in this healthy everyday function occur as a result of PMN subversion by pathological microbial strains, genetic defects or crosstalk with other chronic inflammatory conditions, including cancer and rheumatic disease, and this can provide some avenues for therapeutic targeting of PMN function. In other cases, targeting PMN functions could worsen the disease state. Certain PMN-mediated responses to pathogens, for example Neutrophil Extracellular Traps (NETs), might lead to undesirable symptoms such as pain or swelling and tissue damage/fibrosis. Despite collateral damage, these PMN responses limit pathogen dissemination and more severe damage that would otherwise occur. New data suggests the existence of unique PMN subsets, commonly associated with functional diversification in response to particular inflammatory challenges. PMN-directed therapeutic approaches depend on a greater understanding of this diversity. Here we outline the current understanding of PMNs in health and disease, with an emphasis on the positive manifestations of tissue and organ-protective PMN-mediated inflammation.

## Key Points

- PMNs are constitutively recruited to healthy mucosal tissues
- PMNs prune the commensal biofilm to maintain homeostasis in the oral cavity
- Dysbiosis contributes to hyper-inflammatory PMN responses and periodontal disease
- Targeted therapies to suppress PMN hyper-inflammatory responses are available, but may not be appropriate in cases where PMN-mediated pathogen clearance is required

## Introduction

Polymorphonuclear neutrophils (PMNs) are the most abundant leukocytes in the circulation, and the first cellular responders to tissue injury and infection. Innate immune phagocytic cells are the most ancient immune cells, having evolved ~600 million years ago, in early invertebrate eukaryotes (1). This is in contrast to adaptive immune cells, which evolved ~100 million years later, in early vertebrates. PMNs have evolved under rigorous biological constraints, as a balance is required between the need to respond appropriately to a wide range of threats and the extreme tissue destructive potential of PMN antimicrobial functions. Although PMNs have a relatively short life-span compared to other immune cells, their sheer abundance, innate recognition of damage and infection, and ability to quickly home to relevant sites throughout the body and destroy invading pathogens, attests to their central importance in immune surveillance and protection. In the vast majority of cases PMNs manage to respond suitably to preserve homeostasis and organismal health, however certain pathogenic bacteria have evolved to subvert or evade PMNs (2). Furthermore, dysregulated PMN responses associated with excessive tissue damage (3, 4) are implicated in a wide array of chronic inflammatory diseases including periodontal disease (5, 6), cancer (7–9), sepsis (10, 11), lupus (12), asthma (13), diabetes (14), and rheumatologic diseases (15, 16).

PMNs are generated in the bone marrow by hematopoietic precursor cells and enter the circulation where they can then be recruited across the vascular barrier and into various tissues in response to specific chemotactic and pro-inflammatory signals, a process known as extravasation (17). Once in the tissue, PMNs migrate toward the site of inflammation through highly sensitive detection of a shallow gradient of chemotactic agents and penetrate through the three-dimensional tissue matrix by secretion of tissue-digestive proteases (18). At the site of inflammation, PMNs secrete additional chemotactic signals in order to recruit even more PMNs as well as other protective leukocytes. As PMNs migrate through the tissue, they forge a pathway for migration of subsequently recruited cells and leave a trail of haptokinetic markers (19), which provide cues for directional recruitment of follower cells. PMNs have various tools for neutralizing pathogens including release of toxic enzymes and proteases through degranulation, secretion of reactive oxygen species (ROS), phagocytosis and pathogen killing within the phagosome, and secretion of euchromatic DNA in the form of Neutrophil Extracellular Traps (NETs) (20). These mechanisms are elicited by a wide range of pathogens, which PMNs detect using an array of surface-expressed innate immune receptors, known as pattern-recognition receptors (PRRs) (21). Through these PRRs, PMNs recognize and respond appropriately to a wide range of pathogen-associated molecular patterns (PAMPs) and damage-associated molecular patterns (DAMPs). The quality and magnitude of the PMN response will depend on the nature and abundance of the danger signals present. In addition to surface expression of several families of PRRs, PMNs express complement receptor and low and high-affinity Fc-receptors and thereby act as effectors of complement (22, 23) and antibody mediated inflammation (24). The diverse array of potential PMN activities includes wide-ranging interactions with innate and adaptive immune cells (25), which give PMNs the capacity to influence the progression and outcome of immune responses (26, 27).

PMNs also play an essential role in the resolution of tissue inflammation through the secretion of anti-inflammatory lipid-based molecules, including resolvins (28). PMN secreted NETs can also contribute to resolution through sequestration and turnover of pro-inflammatory factors (29). Therefore, tissue PMNs can act either to amplify or resolve the inflammation. Analysis of the role of PMNs in wound healing suggests that they can either accelerate or impede the process, depending on the specific context (30–33). The presence of specific *Staphylococcus aureus*-derived virulence factors in a wound was shown to promote early PMN recruitment and favorably influence wound healing and closure (34). Also, PMNs have functions that are independent of immune surveillance and protection, including their ability to clear cellular debris (35) and to promote angiogenesis (36).

## Diversity of the PMN Response: Activation States or Subsets?

Classically, PMNs have been considered a terminally differentiated and homogenous population with a limited lifespan (37), low transcriptional activity (38), and an inability to return to circulation after migration to tissues (39). However, there is a growing body of evidence that challenges these assumptions (40). In addition to our observation that a small population of circulating PMNs is primed for rapid recruitment to the tissues (41), other compelling evidence has emerged suggesting the presence of significant PMN heterogeneity (42–48). In the majority of studies, evidence of PMN diversity has been identified in disease states. For example, a low-density PMN population has been shown in blood (49–53), which correlates with clinical manifestations such as vasculitis and synovitis in systemic lupus erythematosus (SLE). Also, myeloid-derived suppressor cells (MDSCs), which are defined by their ability to suppress T-cell proliferation, have been identified in specific inflammatory disease states (54). In cancer, distinct blood PMN subsets have been identified with opposing cancer-related functions and an ability to switch phenotypes (7, 55), which will be discussed in more detail below. Additionally, subsets of PMNs have been identified that (1) reverse transmigrate from the tissue into the circulation (35, 56–58), (2) can migrate to local lymph nodes and perform antigen presentation to T-cells (59, 60), and (3) stimulate marginal zone splenic B-cells to produce diversified immunoglobulins (61).

Although different PMN phenotypes have been identified based on differences in functionality and cell surface marker expression, it is currently unclear as to whether these constitute differentiated subsets of cells, or simply PMNs that have changed their activation state in a developmental manner and in response to specific stimuli. Compounding this issue is the fact that PMN surface markers are exquisitely sensitive and known to be altered by *in vitro* manipulations (6, 62–65). Since PMNs are typically isolated and labeled prior to fixation it is difficult to exclude possible changes in functionality or immunophenotype due to *in vitro* manipulations. In the absence of definitive evidence of PMN differentiation subsets, we must for now conclude that the phenotypes all arise from a common, terminally differentiated PMN progenitor. Furthermore, if the changes in PMN functionality are unidirectional, they could loosely be considered differentiation steps. However, the short life span of PMNs complicates this interpretation since PMN aging and progression toward death/apoptosis, which is also directly tied to functional exertion, is, by definition, a unidirectional process. Although PMNs do not divide and have a limited lifespan, this does not imply that the possible existence of *bona fide* subsets is not of interest. It is difficult to define exactly how much “difference,” for example at the epigenetic or gene expression level, between populations of PMNs, would be enough to delineate a true subset. We suggest that a high threshold of proof should meet the following three criteria: (1) some level of difference in epigenetics or transcriptional output, (2) significant non-plastic difference in functionality, (3) divergent differentiation at some stage of the myeloid lineage.

PMNs are derived from the granulocyte-monocyte progenitor (GMP) in the BM, which also gives rise to monocytes and dendritic cells (66). A recent study using a mass cytometry (CyTOF) approach identified a proliferative precursor cell, downstream of GMP, that gives rise exclusively to PMNs (67). Three unique PMN populations were identified in BM, including the pre-neutrophil cells, immature PMNs and mature PMNs, and these subsets had distinct transcriptional and functional signatures. Functional output, including ROS production, phagocytosis, chemotaxis, and expression levels of granule protein transcripts was increased with PMN maturity level. In addition, the authors found that immature PMNs are mobilized from the BM in tumor bearing mice, which has also been demonstrated by others (7). In the later study, the immature PMNs, which were associated with a T-cell-suppressive, tumor-permissive response, were found in the low density neutrophil (LDN) fraction of density gradients, which would be consistent with reduced granule content, and therefore lower density, expected from an immature PMN. Consistent with this, an early stage committed unipotent PMN progenitor cell was recently identified in BM of mice and humans, which is expanded in cancer, and gives rise to PMNs with T-cell-suppressive, tumor-permissive properties (68). Together these studies indicate that immature PMNs, which result from BM expansion of the PMN progenitor population in response to cancer, have unique pro-tumor functionality.

Another study demonstrated that PMNs mature, or age, in the circulation, in response to microbial exposure, which was mainly characterized by surface shedding of L-selectin (CD62L) (69). *In vivo* aged circulating PMNs had pro-inflammatory properties including heightened integrin activation and an elevated NETotic response. Since the distinct PMN functional outcomes arise from one common differentiated progenitor population in the BM, current knowledge supports a model whereby PMN functional differences occur as a consequence of aging/maturation rather than differentiation to distinct subsets.

## Regulation of PMN Recruitment to Tissues

Circulating PMNs represent a consistent and sizeable destructive potential, which is in reserve, and therefore only fully initiated if and when a significant threat is encountered. In homeostasis or in response to a gradient of potential inflammatory risk, it is important that the initiation and recruitment of PMNs and therefore the magnitude of the PMN response is well-tuned and balanced with respect to the threat. There are a several mechanisms that have evolved to limit PMN responses and restrict unsolicited recruitment. A large fraction of PMNs marginate within the large capillary networks of major organs and are therefore held in reserve for mobilization during inflammation (70). Adhesive capacity or “stickiness” of PMNs is mediated by cell-surface expression and activation of adhesion receptors, including integrins. This function plays a major role in regulating PMN recruitment to activated endothelia. In a recent manuscript, we demonstrate that two differentially primed populations of PMNs, based on surface expression of adhesion receptors, occur in healthy circulation (41). The majority of circulating PMNs are in a *r*esting *s*tate (rsPMNs), characterized by low surface expression of several adhesion receptors that are required for tissue recruitment, including CD11b and CD66a. Approximately 10% of circulating PMNs have elevated basal expression of these markers in health, and are primed for rapid recruitment to inflamed tissues. We found that, within hours of the initiation of acute peritonitis, rsPMNs in the circulation and in the BM undergo induced surface upregulation of adhesion receptors. Priming occurs due to the systemic dissemination of pro-inflammatory factors and in a manner that is proportionate to the severity of the primary infection. Furthermore, the adhesion receptors identified on the surface after PMN priming are granule membrane components and some degree of primary, secondary and tertiary degranulation was associated with this process. These observations have important implications for the mechanism of PMN regulation and how the signals for recruitment are propagated and amplified systemically.

In addition to systemic PMN surface upregulation of adhesion receptors, integrins become activated in response to sub-second triggering through interactions at the inflamed endothelial surface, ensuring that recruitment occurs only at relevant sites of tissue inflammation (71). Integrin-triggering occurs through a catch bond mechanism, whereby engagement with relevant ligands on the endothelial surface induces a conformational change in α_M_-integrin with strong adhesive capacity. Presence of chemoattractants at the apical endothelial surface also combines with integrin-engagement and shear forces to promote PMN transendothelial migration (72). In the absence of pro-inflammatory cues at the endothelial surface, shear forces of blood flow actively restrict PMN responses (73–75), making tissue recruitment unlikely. In one study, patients that had an increased risk of developing post-operative infections were found to have had “sticky” PMNs prior to the operation (76). The “sticky” PMNs had enhanced adhesiveness but reduced migratory properties, suggesting that baseline PMN activation states, which could include genetic or environmental factors, can impact the innate immune reaction during a challenge.

PMNs are classically seen as being inducibly recruited to tissue sites in response to an acute inflammatory event. However, more recently it has come to be appreciated that PMNs are constitutively recruited to healthy mucosal tissues throughout life. This includes the gastrointestinal tract (5, 77), the respiratory tract (78), the reproductive tract (79), and the surface of the eye (80). To a much lesser extent PMNs are also recruited to healthy non-mucosal tissue sites (81), although the role of these “sentinel” PMNs in sterile tissue is unknown.

## The Mouth as an Important Interface Between the Environment and our Tissues; a Potentially Ideal Site for Assessment of PMN Regulation and Function

The oral cavity is an important interface between us and our environment, where there is significant exposure to pathogenic insults, and the constitutive recruitment of large numbers of PMNs to the gingiva serves an important protective function in health (5, 6, 82, 83). The importance of PMN recruitment to the oral cavity is underscored by our findings from studies of hematopoietic stem cell transplantation in mice and humans (84, 85). We found that, following bone marrow engraftment, repopulating PMNs can be detected in the oral cavity, even before they can be detected in the blood. Based on the yield of PMNs in saliva of healthy humans, it is estimated that 50 to 250 million PMNs are recruited to the oral cavity alone each day. Oral PMN load increases 4–10-fold in chronic periodontal disease (PD) (5, 86). These numbers are conservative, considering that a significant number of PMNs die within the gingival tissue, and therefore do not emerge into the saliva (87). Also, oral rinse protocols only recover a portion of the total PMNs therein, while many are swallowed. The sheer number of PMNs that are recruited to the oral cavity indicates that shedding at mucosal surfaces is a major mechanism for their disposal, ensuring the sequestration and depletion of aging/activated PMNs and thus mitigating their potential to cause damage. From this perspective, the commensal biofilm can be seen partly to function as a sink for continual recruitment and turnover of a significant portion of PMNs that are being produced in the bone marrow. Armed-and-ready PMNs are necessary in case of emergency; however, in the absence of emergency, a mechanism for their disposal is required.

Inflammation in the oral cavity can have systemic, whole body, effects. In PD, hyperactive PMN recruitment to the oral cavity is accompanied by increased circulating PMNs (88) and a massive upregulation of pro-inflammatory cytokines in tissue and circulation (89). The oral cavity, with its varied microbial ecosystem, as well as constitutive low-grade PMN mediated effects, is underappreciated in terms of its impact on systemic and overall health. Simultaneously, it represents a vital opportunity to study tissue PMN function in the context of commensal (health) and pathogenic (disease) bacteria, due to easy access to large numbers of PMNs in saliva and gingival crevicular fluid.

## Oral PMNs Prune the Commensal Microbiome and Prevent Dysbiosis

**Figure 1:**
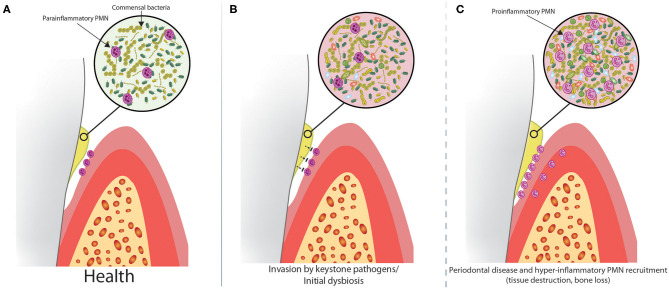
Dysbiosis alters the relationship between biofilm and oral PMNs in a step-wise progression to periodontal disease. **(A)** PMNs are constitutively recruited to the healthy oral cavity through the gingival crevice and limit the growth of the commensal biofilm. Healthy oral PMNs are in a para-inflammatory state, meaning they are not fully activated. **(B)** In the early stages of disease, keystone pathogens suppress oral PMNs, causing increased biofilm growth and dysbiosis. **(C)** At some critical threshold, the dysbiosis becomes severe enough to trigger a massive innate immune response, characterized by recruitment of large numbers of pro-inflammatory PMNs. This leads to tissue destruction and bone loss associated with PD.

Oral PMNs, which are recruited constitutively through the gingival lamina propria and across the junctional epithelium, emerge into the gingival crevicular fluid and saliva, where they perform an essential housekeeping duty to “prune,” and therefore limit the growth of the commensal microbial biofilm (Figure 1A). This continuous function of PMNs in the oral cavity helps to prevent dysbiosis, which would otherwise necessitate an augmented immune response. A wall of PMNs forms on the apical side of the junctional epithelium (90), forming a protective barrier to the gingival biofilm (91). These PMNs restrict biofilm growth through the release of toxic granule contents and ROS production, but cannot phagocytose the large biofilm structure. Although it would be biologically consistent for oral PMNs to preferentially kill pathogenic microbes during early colonization of the otherwise healthy oral cavity, there is evidence that some pathogens can, in fact, evade PMN mediated destruction within the oral cavity (2, 92), which likely accounts for their pathogenic properties.

Sustained oral dysbiosis leads to gingivitis and eventually PD. Dysbiosis associated with specific keystone pathogens is correlated with the onset of PD (2, 93–95). In addition to increased oral PMN levels, we (6, 96, 97) and others (89, 98–100) have shown that oral PMNs of PD patients have a hyper-inflammatory phenotype, characterized by elevated phagocytosis and degranulation, and greater production of ROS and NETs. Despite this, oral PMNs are not able to contain the bacterial infection and instead cause collateral damage to periodontal connective tissue, loss of attachment, and loss of alveolar bone leading to tooth loss (98). Through careful analysis of PMN surface markers of activation, we showed that in health, oral PMNs have a reduced, para-inflammatory activation state compared to the pro-inflammatory phenotype of oral PMNs from patients with PD (6). This indicates that PMNs can shift gears and respond differently to non-pathogenic commensal organisms or pathogenic organisms within the oral environment.

Although the self-regulating ecological attributes of commensal microbial biofilm communities are highly sophisticated, these biofilms are in a constant state of flux due to environmental exposure and other factors. PMNs can help to limit the relative abundance of specific microbial strains and therefore shape the constituency of the biofilm. A model for progression of PD pathogenesis is presented in Figure 1. Subversion or avoidance of PMN mediated destruction by an invading keystone pathogen could potentially impact the ability of PMNs to limit growth of both the invading pathogens and the commensal organisms, eventually reaching a hypothetical threshold where the dysbiotic microenvironment triggers a magnified PMN-mediated inflammatory response (Figures 1B,C). This model is supported by manifestations in the absence of normal PMN function. PMN recruitment is defective in leukocyte adhesion deficiency Type-I (LAD-I), and this is, seemingly paradoxically, associated with increased inflammation within the gingival tissues (101, 102). While the altered biofilm associated with the absence of PMNs did not penetrate into the diseased gingival tissues, microbial factors did penetrate into the tissues and drive T-cell and epithelial cell-derived IL-17 and IL-23, which induced gingival inflammation associated with symptoms of periodontal disease progression, including bone loss and edentation. In a situation where PMNs are suppressed by invading keystone pathogens, or neutropenia following chemotherapy, oral dysbiosis might trigger a similar IL-17/IL-23 axis, but could also cause a hyperinflammatory PMN recruitment cascade which might supersede the initial suppression. Although the clinical appearances of periodontal tissue destruction in the absence of functional PMN-mediated protection might appear to be similar to that seen in patients with PD, the latter being mediated in part by hyperfunctional PMNs, the underlying pathophysiological processes of the two are quite different. In relation to conditions defined by reduced PMN function, the fact that bacteria can actually invade tissues becomes more important in that these bacteria not only stimulate other inflammatory pathways, as alluded to above, but might also cause direct damage to tissues by way of bacterial virulence factors (e.g., bacterial collagenases).

## Ocular PMNs

The surface of the eye is somewhat analogous to the mouth, in that it has its own commensal biofilm and shows constitutive recruitment of PMNs. PMNs accumulate at the healthy ocular surface primarily in the closed eye environment, during sleep (80). PMNs from the closed eye have a suppressed ability to respond to *in vitro* stimuli (103, 104), suggesting that some aspect of the ocular environment in the closed eye limits full-scale activation of PMNs, which might serve to protect the corneal surface from ROS and granule proteins, while maintaining the ability of PMNs to protect the eye from pathogens. The anti-inflammatory tear protein, lactoferrin, was shown to partly suppress blood PMN responses to *in vitro* stimuli (103). Furthermore, evidence indicates that NET formation on the ocular surface during sleep could have anti-inflammatory properties, with NET aggregates and associated pro-inflammatory factors being ejected at the corner of the eye upon waking (105). During a corneal injury, PMN recruitment to the eye is greatly increased (106).

## Defective PMN Responses

Although the vast majority of PMN mediated inflammatory events exhibit a graded and appropriate response that limits and contains the spread of pathogens effectively, defective PMN responses can occur for reasons that are either genetic or environmental, with serious consequences to human health. Defects in production, release, recruitment, or function of PMNs are all associated with increased risk of infection and death (107–109). Neutropenia, or low circulating PMN counts, is defined as an absolute PMN count of ≤ 500/mm^3^ and, most often, is the result of a genetic disorder or chemotherapy (110–112). Neutropenia is commonly treated with granulocyte colony-stimulating factor (G-CSF) or granulocyte-macrophage colony-stimulating factor (GM-CSF) (113), which mobilize and prime PMNs (41, 114, 115).

In addition to neutropenia, several rare genetic defects of PMN function exist. Chronic granulomatous disease (CGD), in which PMNs engulf but cannot kill microorganisms, can leave an individual more susceptible to frequent infections including, pneumonia, infections in the lymph nodes and abscesses in the skin, liver and other organs (107). Chédiak-Higashi disease, caused by a defect in PMN primary granules, is also manifested clinically by recurrent infections, severe periodontal disease and early death (116). Leukocyte adhesion deficiencies (LAD) are due to mutations that cause defective β2-integrin (CD18) function (LAD I), or defective glycosylation of selectin family adhesion receptors (LAD II). This leads to the formation of PMNs that cannot adhere to vascular endothelium and thus cannot migrate out of blood vessels into areas of infection (117, 118), causing elevated blood PMN counts and severe bacterial infections, especially within the mouth and gastrointestinal tract. LAD has been treated successfully with bone marrow transplants.

Papillon-Lefèvre syndrome is a rare genetic disease of chronic non-resolving inflammation characterized by a loss of PMN NETotic ability and serine protease activity, resulting in impaired microbicidal functions (119). PMNs in these patients are competent at cytokine production and ROS generation and are recruited relentlessly to sites of tissue inflammation, but fail to successfully limit microbial growth. The resulting “frustrated” inflammation causes the development of severe periodontal disease leading ultimately to the loss of all teeth (deciduous and secondary). Thus, a defect in certain PMN functions can paradoxically result in compensatory hyper-inflammation through other PMN functions, as a result of the non-clearance of pathogens. PMN defects often manifests in the oral cavity, which is a major interface between us and our microbe laden environment, as noted above.

## PMNs are Subverted in Cancer

Cancerous cells are derived from the human body and therefore are “self,” however they do present specific cancer associated patterns (CAPs) that are recognized and targeted by both innate and adaptive immune cells. The innate immune system can recognize unique CAPs to prevent tumor growth and metastasis (120). However, the role of PMNs in cancer is complex, and they are not only capable of killing tumor cells but also promoting tumor growth (121, 122). Although PMNs have anti-tumor potential (7), tumors can subvert PMN function (8, 54, 55), including NET formation (123), to promote tumor growth. PMNs can also contribute to metastasis (9, 124). Significant diversity of PMN populations has been identified based on gene expression analysis in cancer. In both human non-small-cell lung cancer (NSCLC) patients and a mouse model of NSCLC, up to 6 different tumor associated PMN populations were demonstrated (125), based on distinct patterns of transcript expression. In a mouse model of breast cancer, three distinct circulating PMN populations were described, with significant functional plasticity (7). The authors showed that early in tumor progression, PMNs help to clear cancer cells from the circulation, however, at later stages, tumor associated neutrophils (TANs) switch to a pro-tumor, immunosuppressive phenotype (7, 55). One recent study demonstrated that PMNs exhibit pro-tumor attributes in a hypoxic tumor microenvironment, and oxygenation of the tumor induced these PMNs to revert to their tumor killing phenotype (126). As described in the section “Diversity of the PMN Response: Activation States or Subsets?”, immature T-cell-suppressive PMNs, called PMN-myeloid derived suppressor cells (PMN-MDSCs), are associated with the tumor-permissive PMN phenotype in cancer (54). Chemotherapeutic treatment causes neutropenia and these patients are at risk of developing severe infections (112). It seems reasonable to predict that the treatment might partly be effective based on the reduction of tumor-permissive PMNs, to our knowledge, this avenue has not been explored.

Manipulating PMN responses could be the basis of an effective potential cancer immunotherapy approach, the goal of which would be to restore the pro-inflammatory function of PMNs and direct them toward tumor associated molecular patterns (127). A recent study has shown that pores in the endothelial barrier, formed by extravasating PMNs, improve the delivery of liposomes to tumors (128), and therefore could contribute to efficacy in new cancer therapy approaches that rely on delivery of drugs inside liposomes.

In addition to the ability of tumors to induce pro-tumor TANs, oral cancers could be subject to the effects of PMNs that occur naturally and constitutively in the oral cavity or as a result of PD. Elevated oral PMNs are directly correlated with reduced survival and increased recurrence of oral squamous cell carcinoma (OSCC) (129). Co-culture experiments demonstrated that PMN factors increased the invasive potential of OSCC cells (130). Accordingly, PD, which is associated with massively upregulated PMN recruitment to the oral cavity, is also a major risk factor associated with oral cancers (131, 132). In addition to increased oral PMN counts in PD as discussed previously, evidence suggests that these PMNs exhibit an immunosuppressive phenotype, based on production of IL-10 (133), which is similar to the phenotype exhibited by tumor-permissive PMNs. PMNs could also contribute to cancer progression through matrix remodeling and angiogenesis (134), and the nature of the chronically inflamed microenvironment of the gingival wound in PD, to which PMN-mediated tissue damage contributes greatly, can act as the ideal niche for tumor seeding and progression. Finally, NETs, which are highly elevated in PD (6), have been implicated in cancer progression (9, 135) and metastasis (136–138), and measuring NETs has been proposed as a method to determine increased risk of metastasis in head and neck squamous cell carcinoma (HNSCC) (139).

## Potential Therapeutic PMN-Specific Interventions

Pathological or auto-inflammatory PMN responses occur in various disease states, and can cause significant tissue damage and tissue liquefaction in extreme cases (4, 140). In these instances, PMN-targeted therapies are possible (141). Current PMN targeted therapies include inhibition of PMN recruitment, suppression of PMN effector functions and enhancement of PMN apoptosis (142, 143).

Sepsis, a common cause of death in hospital intensive care units, is caused by severe polymicrobial infections, causing cytokine storm (144) and massive systemic activation of PMNs, followed by PMN paralysis (145). Highly activated PMNs in circulation develop increased membrane rigidity resulting in sequestration in the capillary beds (146–148). These PMNs fail to transmigrate and contribute to ischemic injury and organ damage. Damage to the vasculature causing loss of tissue perfusion and oxygen delivery results in death due to the failure of vital organs such as the kidneys, liver, heart, and lungs (149–151). Limited effective treatment options are available for sepsis (152, 153), and early intervention is critical (154). Early PMN recruitment to resolve the initial infection (155, 156) and early administration of antibiotics (157) are both associated with increased survival rate. Also, pre-administration of probiotics (158) and mesenchymal stem cell transfusion (159) have both shown efficacy in reducing sepsis mortality in mice by suppressing infection and inflammation. Sepsis often results in acute respiratory distress syndrome (ARDS), with excessive PMN recruitment to the lung alveoli associated with increased mortality (160). In a pig model of ARDS, suppression of PMN proteases, including matrix metalloproteinase-2 (MMP-2), MMP-9 and elastase, effectively suppressed the development of sepsis and ARDS (161). Although suppression of PMNs is one potential treatment option for sepsis, it is complicated by the fact that PMNs play both protective and destructive roles (11). Furthermore, suppression of the inflammatory PMN response to infectious agents, including bacteria, viruses, and parasites, will inevitably lead to increased host susceptibility. This is illustrated by the observation that mutant mice lacking TLR4, a receptor for bacterial LPS, are resistant to septic shock, but more prone to infection by gram-negative bacteria (162). The timing of treatment in sepsis is an important factor to consider, as there could be a therapeutic window where PMN suppression can avoid certain aspects of the hyperinflammatory response such as vascular damage, and simultaneously keep the PMNs on track to deal with the infection after. PMN suppressive therapies might be effective in combination with antibiotic treatment. PMNs can also act as a biomarker for the diagnosis of sepsis. Surface upregulation of the high-affinity Fc-receptor, CD64, on circulating PMNs, is used clinically as a marker of sepsis (163, 164).

Gout is a painful arthritic disease caused by the recruitment of PMNs to the inflamed joint in response to the build-up of crystals of the natural metabolite, monosodium urate (MSU). Although NSAIDs are currently used as the standard treatment for acute gout, the microtubule depolymerizing agent, colchicine, has been used for this purpose for centuries (165). Colchicine directly inhibits PMN responses to MSU crystals that cause gout, including adhesion, chemotaxis, recruitment, ROS production, and activation of the NLP3 inflammasome (166–169). Interestingly, gall stones, which are also made of crystalized MSU, require NETs in order to form (170), suggesting that PMN targeted therapies could be useful for this common disease.

Antibodies can be used to block cytokines, PMN surface epitopes or epithelial-expressed adhesive ligands to block PMN recruitment to tissues. For example, Anti-TNF and anti-IL17 injection into arthritic joints are an effective method to suppress PMN-mediated inflammation in rheumatoid arthritis (171). Antibodies that block the PMN expressed surface carbohydrate Sialyl Lewis^x^ (CD15) reduce PMN chemotaxis and tissue recruitment (172). ICAM-1 expressed on intestinal epithelial cells acts to recruit PMNs to the gut and reduce PMN apoptosis (30) and is, therefore, a potential target for antibody-mediated therapy in gut inflammation.

Targeting NET production is a relatively new field with significant potential in the treatment of specific PMN-mediated pathologies. Auto-antibodies against endogenous NETs have been implicated in the pathogenesis of RA and SLE (173). In SLE, NETs were found to trigger macrophages in a feedforward loop, stimulating further NET production, which could contribute to flares (174). Therapies targeting NETs could suppress macrophage activation and limit immune complex formation and therefore suppress downstream activation of adaptive immune responses in SLE (175). Recently it has been shown that aggregated NETs contribute to resolution of inflammation by sequestering and degrading pro-inflammatory factors (29), which should be kept in mind in any therapeutic intervention targeting NETs.

Some of the anti-inflammatory effects of cannabis are likely through the suppression of PMN function. PMNs express the cannabinoid receptor (CB2) (176) and evidence suggests that cannabinoids suppress PMN function including cell migration, production of ROS and TNF-α production (177). *In vivo*, inhibition of the CB2 receptor suppressed PMN-mediated tissue damage in a mouse model of myocardial ischemia/reperfusion injury (178), and mice that lack CB2 expression exhibit exacerbated PMN recruitment during infection (179).

Natural lipid factors, including resolvins, protectins, and maresins, which are pro-resolution as opposed to anti-inflammatory, have the potential to suppress over-exuberant or unwanted inflammation (180). Resolvin D2 (RvD2) was shown to reduce the innate immune response and alveolar bone loss in a mouse model of *P. gingivalis*-induced periodontitis (181).

Other factors with potential therapeutic PMN-suppressive functions include Benzyloxycarbonyl-proline-prolinal (ZPP) (182), the endogenous glucocorticoid annexin A1 (183), galectin 1 (184), and carbon monoxide (185), hydrogen sulfide (186), and nitrous oxide gas (187).

PMN responses can contribute to pain and other undesirable symptoms. However, cessation of PMN function might interrupt the body's natural defenses and therefore worsen prognosis, and could lead to alternative consequences that are more severe. A recent mouse study demonstrated that retinal damage and infection with *P. aeruginosa* resulted in the recruitment of PMNs to the corneal surface of the eye, and generation of a layer of NETs that acted as a barrier to the infecting biofilm (188). Although the resulting inflammation caused significant keratitis-associated damage to the eye, the alternative, demonstrated in PAD4^−/−^ mice that are not able to form NETs, was the dissemination of the bacterial infection into the brain, a decidedly more severe outcome. Similarly, NET production in the heart causes organ damage associated with old age (189), and PAD4^−/−^ mice lack signs of age-related fibrosis of the heart. However, in the absence of PMN mediated surveillance and NETosis, other negative cardiovascular consequences and infections would likely occur. Therefore, aging-associated tissue fibrosis is an undesirable consequence of the natural protective functions of PMNs.

## PMNs are a Nexus for Simultaneously Occurring Inflammatory Triggers

Several chronic inflammatory diseases including heart disease, rheumatoid arthritis, and diabetes are reciprocally linked with PD (190–193). In a recent longitudinal study, following a cohort of 161,286 subjects over a 10.5 year period, a direct association was found between reduced oral hygiene and severity of PD with increased incidence of atrial fibrillation and heart failure (194). The mechanisms underlying the crosstalk between two independent inflammatory conditions occurring at different loci within the body are unknown, however PMNs are a good candidate cell type that could contribute, since PMN priming and mobilization occurs systemically. Supporting the link between oral inflammation and systemic PMN responses, we found increased priming of PMNs in the circulation 1 week after cessation of oral hygiene practices, using a human model of experimental gingivitis (41). Another study showed that the presence of PD in diabetic patients exacerbated the suppression of apoptosis of circulating PMNs compared to diabetes alone (195). Although PMNs generally are recruited across the inflamed endothelium in a highly targeted manner to respond to an acute or chronic threat, what happens when a patient has an additional underlying inflammatory complication or comorbidity? It seems reasonable to speculate that PMNs that are generated, demarginated or primed in response to an inflammatory challenge will raise the innate immune highwater mark and therefore lower the threshold of immune responses to other challenges (196). In support of this, mice with underlying chronic inflammation including colitis, diabetes and lung inflammation showed increase PMN recruitment to an unrelated secondary peritoneal site (197). In a pig model of acute lung injury, a second “hit” introduced by injection of low dose LPS caused more severe organ damage, which was associated with hyper-inflammatory PMN recruitment and secretion of MMP-9 and elastase (198). PMN therapeutic approaches could be effective in short-circuiting the interaction between two parallel inflammatory conditions.

## Role of PMNs in Aging

Collateral damage due to inflammation contributes to the aging process (189, 199–201) and is a major contributor to pathological disease progression in old age (202). The sheer abundance of PMNs and their tissue destructive properties suggest that cumulative PMN-mediated effects are likely to play an important role in the aging process, including contributions to chronic and acute disease, tissue fibrosis and periodontal bone loss. Aging-associated accumulation of DAMPs is one mechanism whereby PMNs could contribute to chronic low-grade inflammation in the elderly. Furthermore, changes in the immune system during aging, called inflammaging, are characterized by reduced PMN function. With old age PMN functions including chemotaxis (203), phagocytosis (204), and NETosis (205, 206) are reduced. In spite of this, total WBC and PMN counts (207), levels of pro-inflammatory factors in the circulation, and the risk of severe and deadly hyper-inflammatory innate immune responses to acute challenges (208) are all increased.

**Figure 2:**
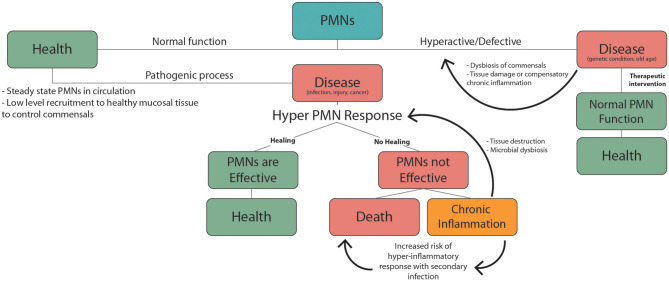
PMNs responses during health and disease. Normal PMN function includes low level recruitment to healthy mucosal tissues to limit growth of the commensal biofilm. Pathogenic factors including infection, injury, and cancer trigger inflammatory PMN responses. In the case of an effective response homeostasis/health is achieved. If not this can lead to chronic inflammation or death. Non-resolving chronic inflammation can produce PMN mediated tissue damage and dysbiosis of commensal microbes, which leads to further exacerbation of inflammation. Underlying chronic inflammatory conditions, which are common in the elderly, increase the risk of severe hyper-inflammatory responses to an unrelated secondary inflammatory trigger. These hyper-inflammatory PMN-mediated responses including sepsis and ARDS, and are associated with high mortality. Hyperactive or defective inflammatory responses can also occur due to genetic conditions or old age, leading to dysbiosis, chronic inflammation, and tissue damage. PMN targeted therapies can help mitigate these defects to promote normal PMN function and health.

Suppressed innate immune function in old age causes the elderly to be more susceptible to infectious disease. This is confounded by the fact that many elderly people have underlying health conditions to begin with. From this perspective it is helpful to consider changes in PMN responses during aging in the context of the double hit model of inflammation discussed in the previous section. From this perspective, chronic low-grade inflammation associated with common health complications of the elderly could produce dysfunctional innate immune responses and increased mortality during an acute health emergency (Figure 2). This has recently been highlighted by the increased mortality rates associated with COVID-19 infection in the elderly. Ten to 15% of COVID-19 patients are highly susceptible to severe outcomes including pneumonia, acute respiratory distress syndrome (ARDS) and septic shock (209), and this risk is much higher in the elderly population. These adverse responses that are responsible for the high mortality rate associated with COVID-19 infection are characterized by excessive recruitment of PMNs to the lungs and other vital organs. Patients suffering from acute respiratory distress syndrome (ARDS) due to COVID-19, show massive PMN recruitment and NETosis in the lungs (210), and elevated NETotic DNA in the circulation (211).

## Concluding Remarks

In health, PMNs are constitutively recruited to mucosal tissues, including the oral cavity, gastrointestinal, respiratory and reproductive tracts and the ocular surface, and a lesser extent to naive sterile tissues throughout the body. They also help to contain transient bacteremias (212) and can be quickly recruited in large numbers to sites of infection throughout the body. Through their innate immune function and as effector cells of the adaptive immune response, PMNs consistently protect the host organism from existential threats. Despite this, PMN responses tend to be recognized mainly due to their tissue destructive effects.

Some of the consequences of PMN function that might be considered undesirable are actually protective and preferable to the alternative. In periodontal disease, for example, a massive influx of PMNs into the oral cavity and overt inflammation of the gingiva results in bone resorption and tooth loss. Prior to the development of modern dentistry, a relatively new phenomenon, these adaptive tissue reactions were desirable outcomes, since tooth exfoliation at sites of advanced periodontal bone loss seals off openings through the mucosa. This functions to protect from the systemic and local effects of chronic gingival inflammation and limits the occurrence of severe invasive lesions such as osteomyelitis. Consistent with the protective effects of tooth exfoliation, PMN activation is suppressed in the oral cavity of edentulous patients (213).

PMNs are the most abundant leukocytes and yet they are relatively understudied. This is partly due to their highly volatile nature and experimental intractability. Future research using new cutting-edge approaches, such as single cell RNAseq, mass cytometry and intravital microscopy, will help to develop a deeper understanding of PMN subset diversity and new opportunities for therapeutic intervention.

## Author Contributions

NF wrote the manuscript and collaborated to produce the figure. NT collaborated to produce the figure and edited the document. MG, HT, and CM provided guidance, edited, and helped write the manuscript. All authors contributed to the article and approved the submitted version.

## Conflict of Interest

The authors declare that the research was conducted in the absence of any commercial or financial relationships that could be construed as a potential conflict of interest.
